# The genome sequence of the Oak Rustic, *Dryobota labecula *(Esper, 1788)

**DOI:** 10.12688/wellcomeopenres.19541.1

**Published:** 2023-07-21

**Authors:** Peter W.H. Holland, University of Oxford and Wytham Woods Genome Acquisition Lab, Darwin Tree of Life Barcoding collective, Wellcome Sanger Institute Tree of Life programme, Wellcome Sanger Institute Scientific Operations: DNA Pipelines collective, Tree of Life Core Informatics collective, Darwin Tree of Life Consortium

**Affiliations:** 1 University of Oxford, Oxford, England, UK

**Keywords:** Dryobota labecula, Oak Rustic, genome sequence, chromosomal, Lepidoptera

## Abstract

We present a genome assembly from an individual female *Dryobota labecula* (the Oak Rustic; Arthropoda; Insecta; Lepidoptera; Noctuidae). The genome sequence is 767.0 megabases in span. Most of the assembly is scaffolded into 32 chromosomal pseudomolecules, including the W and Z sex chromosomes. The mitochondrial genome has also been assembled and is 15.38 kilobases in length. Gene annotation of this assembly on Ensembl identified 18,924 protein coding genes.

## Species taxonomy

Eukaryota; Metazoa; Eumetazoa; Bilateria; Protostomia; Ecdysozoa; Panarthropoda; Arthropoda; Mandibulata; Pancrustacea; Hexapoda; Insecta; Dicondylia; Pterygota; Neoptera; Endopterygota; Amphiesmenoptera; Lepidoptera; Glossata; Neolepidoptera; Heteroneura; Ditrysia; Obtectomera; Noctuoidea; Noctuidae; Xyleninae; *Dryobota*; *Dryobota labecula* (Esper, 1788) (NCBI:txid2964684).

## Background

The Oak Rustic, *Dryobota labecula,* is a small moth in the family Noctuidae with a sparsely scattered distribution across Europe, from Portugal to Greece ( GBIF Secretariat, 2022). The species is found primarily in woodland and forested regions around the Mediterranean coast ( GBIF Secretariat, 2022). Small numbers of records from northern Europe have been attributed to dispersing or vagrant individuals, plus recently established populations. In Britain, the moth was first recorded from the Isle of Wight in 1999 and on the mainland in 2005 ( Randle *et al.*, 2019); this followed discovery in Jersey and Guernsey in the early 1990s ( Burrow, 1996). The moth appears to be spreading very slowly, with a small number of adults recorded each year from southern counties of England ( Lepidoptera UK, 2023; NBN Atlas Partnership, 2021; Randle *et al.*, 2019).

The adult moth has dusky brown forewings with a pale cream or buff reniform stigma, and is on the wing in late autumn. The eggs overwinter before larvae hatch in spring ( Wagner, 2023). Larvae have been recorded feeding on leaves of holm or evergreen oak ( *Quercus ilex*) or on kermes oak ( *Quercus coccifera*) ( Randle *et al.*, 2019; Wagner, 2023). The larva has striking colouration, with chestnut brown on the dorsal side, sharply demarcated from a scalloped lateral band of yellow-green; it has been suggested to resemble an oak catkin in appearance ( Shaw, 2015). After forming a cocoon in soil, larvae rest for several weeks before pupation ( Wagner, 2023).

A complete genome sequence for *Dryobota labecula* will facilitate research into molecular adaptations to oak feeding and contribute to the growing set of genomic resources for studying the evolution of Lepidoptera.

## Genome sequence report

**Figure 1.**
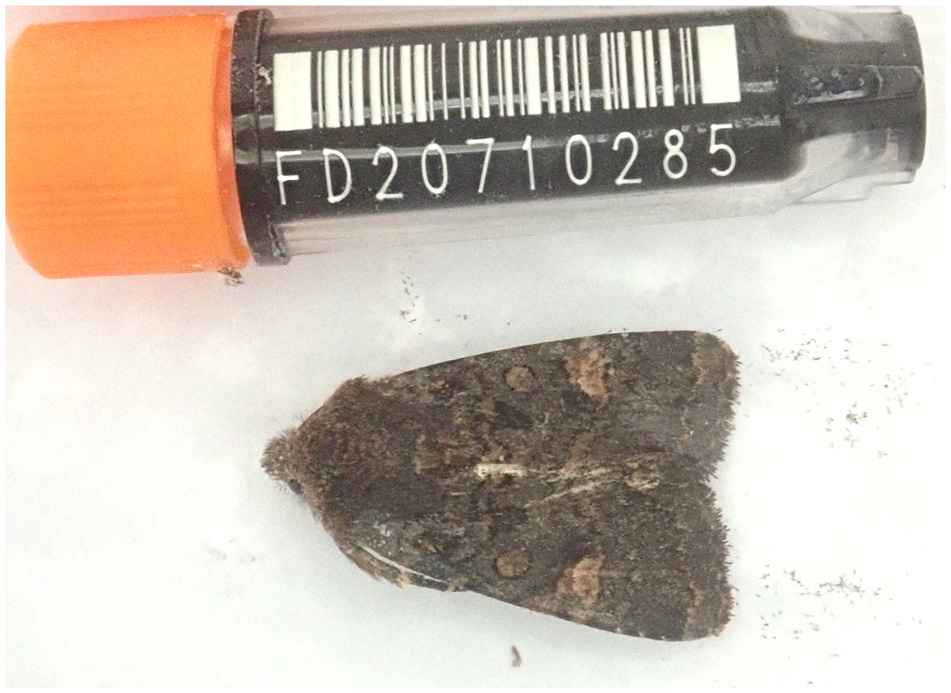
Photograph of the *Dryobota labecula* (ilDryLabe1) specimen used for genome sequencing.

The genome was sequenced from a female *Dryobota labecula* ( Figure 1) collected from Wytham Woods, Oxfordshire, UK (51.77, –1.34). A total of 31-fold coverage in Pacific Biosciences single-molecule HiFi long reads was generated. Primary assembly contigs were scaffolded with chromosome conformation Hi-C data. Manual assembly curation corrected 16 missing joins or mis-joins and removed 1 haplotypic duplication, reducing the scaffold number by 12.

**Figure 2.**
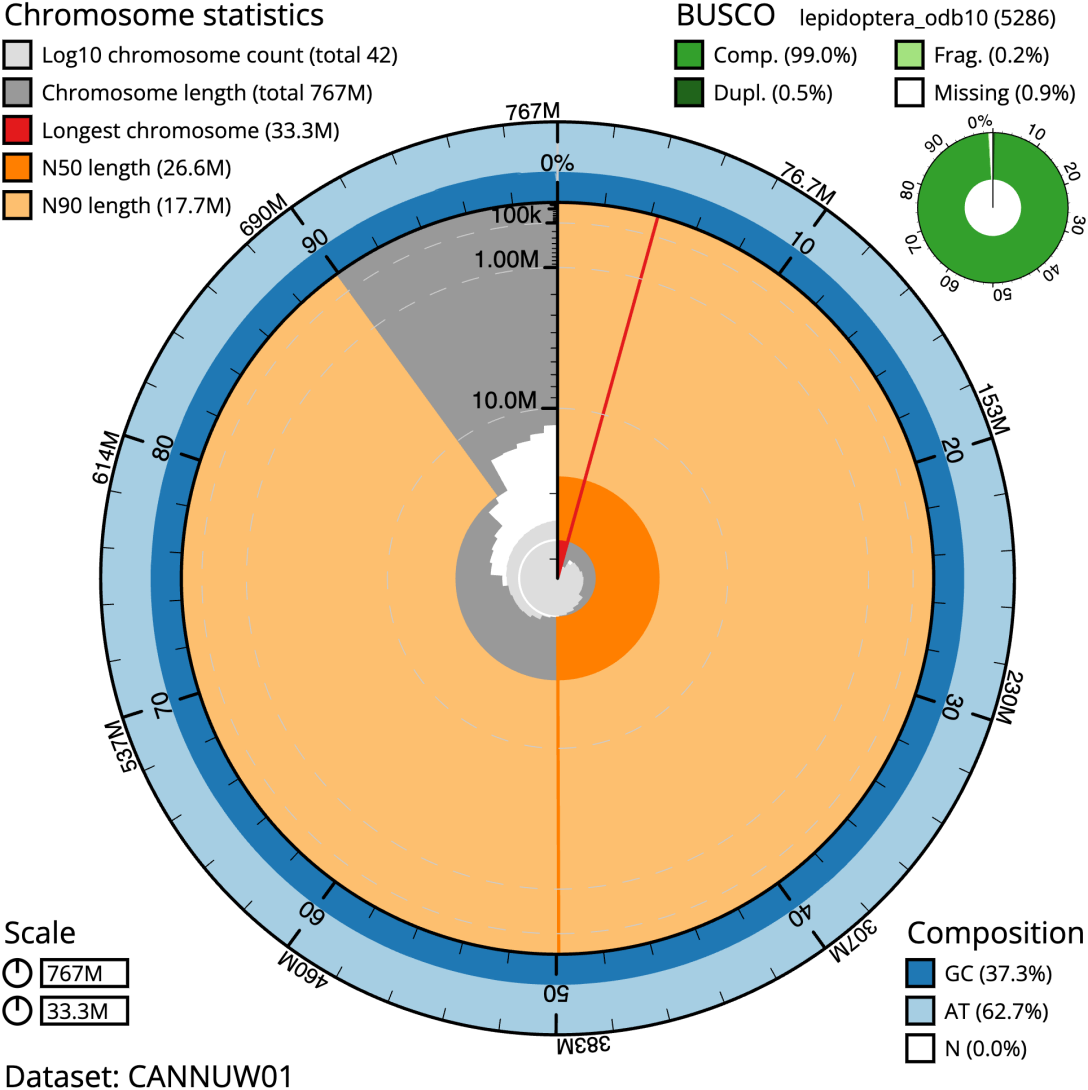
Genome assembly of *Dryobota labecula*, ilDryLabe1.1: metrics. The BlobToolKit Snailplot shows N50 metrics and BUSCO gene completeness. The main plot is divided into 1,000 size-ordered bins around the circumference with each bin representing 0.1% of the 766,991,931 bp assembly. The distribution of scaffold lengths is shown in dark grey with the plot radius scaled to the longest scaffold present in the assembly (33,326,185 bp, shown in red). Orange and pale-orange arcs show the N50 and N90 scaffold lengths (26,640,437 and 17,693,659 bp), respectively. The pale grey spiral shows the cumulative scaffold count on a log scale with white scale lines showing successive orders of magnitude. The blue and pale-blue area around the outside of the plot shows the distribution of GC, AT and N percentages in the same bins as the inner plot. A summary of complete, fragmented, duplicated and missing BUSCO genes in the lepidoptera_odb10 set is shown in the top right. An interactive version of this figure is available at https://blobtoolkit.genomehubs.org/view/ilDryLabe1.1/dataset/CANNUW01/snail.

The final assembly has a total length of 767.0 Mb in 41 sequence scaffolds with a scaffold N50 of 26.6 Mb ( Table 1). Most (99.94%) of the assembly sequence was assigned to 32 chromosomal-level scaffolds, representing 30 autosomes and the W and Z sex chromosomes. Chromosome-scale scaffolds confirmed by the Hi-C data are named in order of size ( Figure 2– Figure 5; Table 2). While not fully phased, the assembly deposited is of one haplotype. Contigs corresponding to the second haplotype have also been deposited. The mitochondrial genome was also assembled and can be found as a contig within the multifasta file of the genome submission.

**Table 1.** Genome data for *Dryobota labecula*, ilDryLabe1.1.

Project accession data		
Assembly identifier	ilDryLabe1.1	
Species	*Dryobota labecula*	
Specimen	ilDryLabe1	
NCBI taxonomy ID	2964684	
BioProject	PRJEB57678	
BioSample ID	SAMEA110451497	
Isolate information	ilDryLabe1, female	
Assembly metrics *		*Benchmark*
Consensus quality (QV)	67.5	*≥ 50*
*k*-mer completeness	100%	*≥ 95%*
BUSCO **	C:99.0%[S:98.5%,D:0.5%], F:0.2%,M:0.9%,n:5,286	*C ≥ 95%*
Percentage of assembly mapped to chromosomes	99.94%	*≥ 95%*
Sex chromosomes	Z and W chromosomes	*localised homologous pairs*
Organelles	Mitochondrial genome assembled	*complete single alleles*
Raw data accessions		
PacificBiosciences SEQUEL II	ERR10499361	
Hi-C Illumina	ERR10501027	
Genome assembly		
Assembly accession	GCA_947523025.1	
*Accession of alternate haplotype*	GCA_947522625.1	
Span (Mb)	767.0	
Number of contigs	131	
Contig N50 length (Mb)	10.2	
Number of scaffolds	41	
Scaffold N50 length (Mb)	26.6	
Longest scaffold (Mb)	33.3	
Genome annotation		
Number of protein-coding genes	18,924	
Number of gene transcripts	19,099	

* Assembly metric benchmarks are adapted from column VGP-2020 of “Table 1: Proposed standards and metrics for defining genome assembly quality” from ( Rhie *et al.*, 2021). ** BUSCO scores based on the lepidoptera_odb10 BUSCO set using v5.3.2. C = complete [S = single copy, D = duplicated], F = fragmented, M = missing, n = number of orthologues in comparison. A full set of BUSCO scores is available at https://blobtoolkit.genomehubs.org/view/ilDryLabe1.1/dataset/CANNUW01/busco.

**Figure 3.**
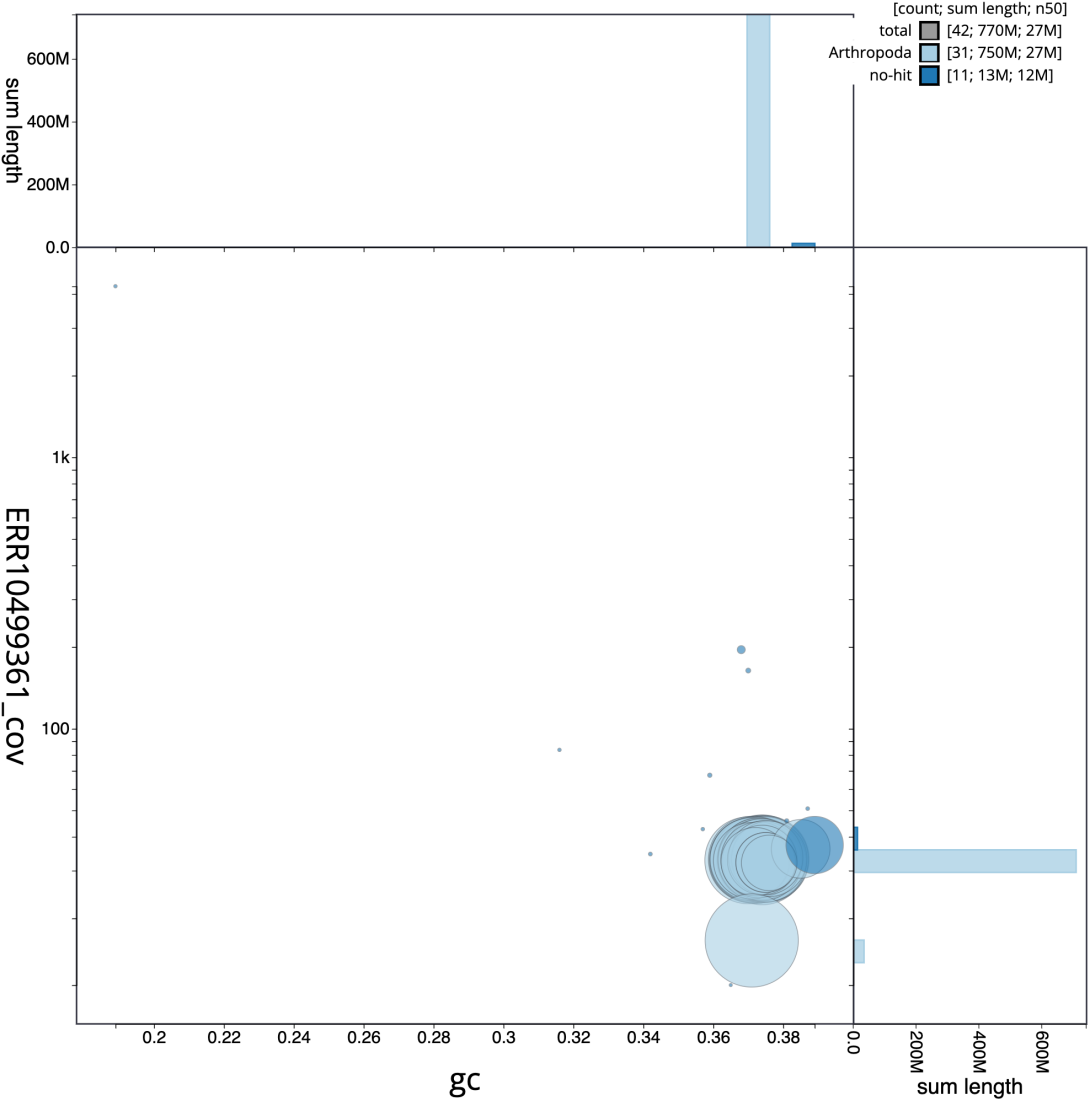
Genome assembly of *Dryobota labecula*, ilDryLabe1.1: BlobToolKit GC-coverage plot. Scaffolds are coloured by phylum. Circles are sized in proportion to scaffold length. Histograms show the distribution of scaffold length sum along each axis. An interactive version of this figure is available at https://blobtoolkit.genomehubs.org/view/ilDryLabe1.1/dataset/CANNUW01/blob.

**Figure 4.**
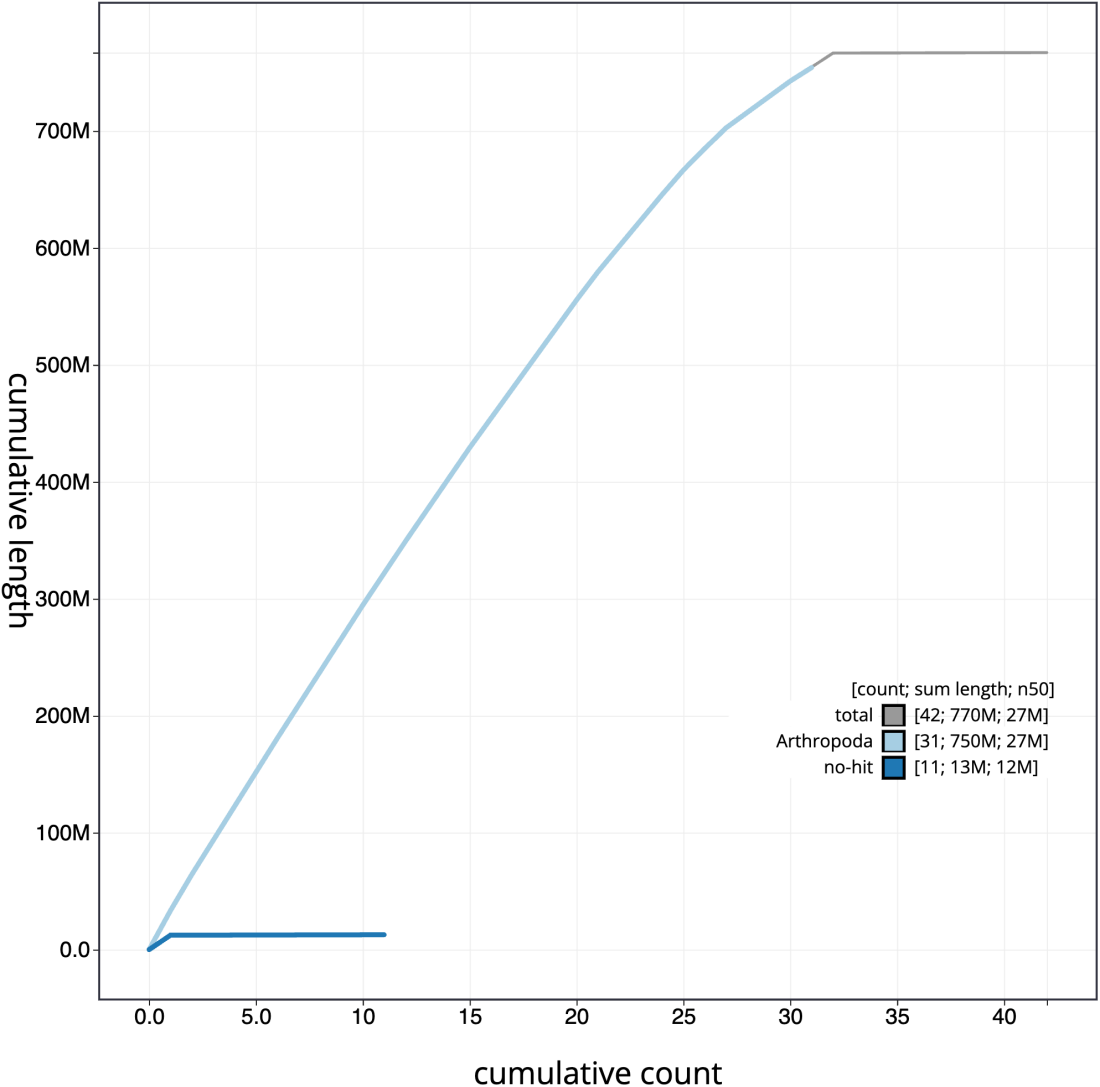
Genome assembly of *Dryobota labecula*, ilDryLabe1.1: BlobToolKit cumulative sequence plot. The grey line shows cumulative length for all scaffolds. Coloured lines show cumulative lengths of scaffolds assigned to each phylum using the buscogenes taxrule. An interactive version of this figure is available at https://blobtoolkit.genomehubs.org/view/ilDryLabe1.1/dataset/CANNUW01/cumulative.

**Figure 5.**
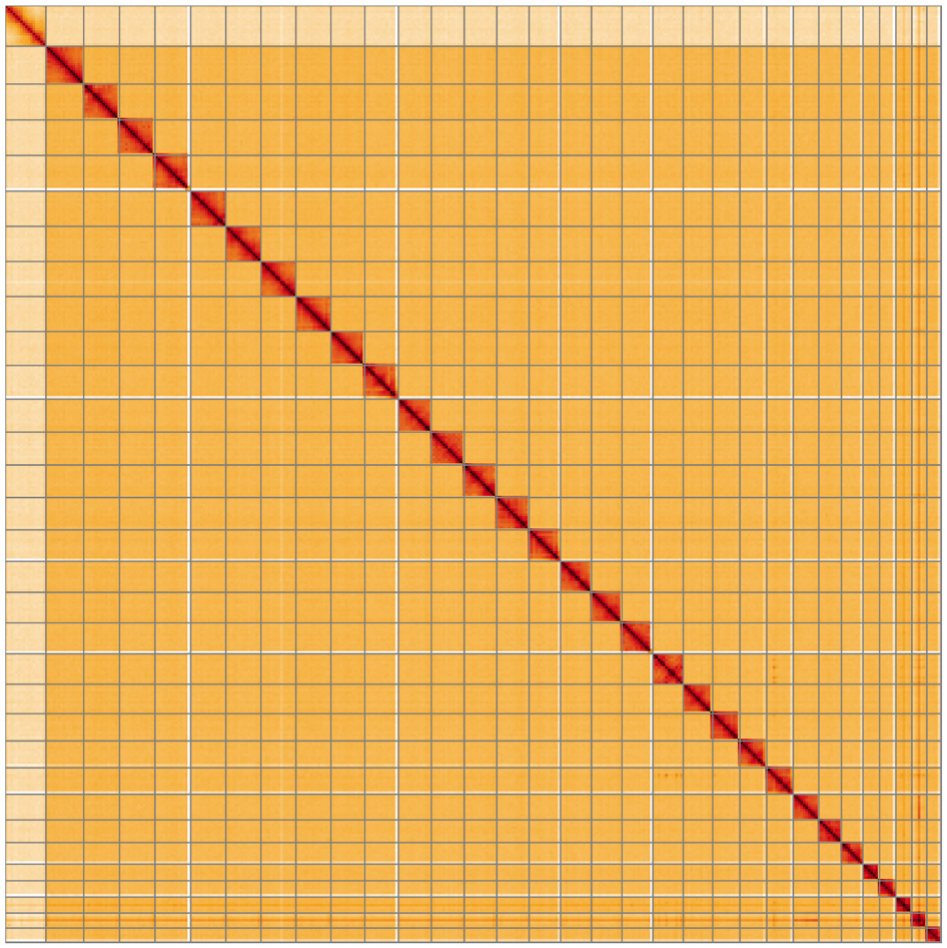
Genome assembly of *Dryobota labecula*, ilDryLabe1.1: Hi-C contact map of the ilDryLabe1.1 assembly, visualised using HiGlass. Chromosomes are shown in order of size from left to right and top to bottom. An interactive version of this figure may be viewed at https://genome-note-higlass.tol.sanger.ac.uk/l/?d=dn_Mp2ihR3GkhmKW_dvmVw.

**Table 2.** Chromosomal pseudomolecules in the genome assembly of *Dryobota labecula*, ilDryLabe1.

INSDC accession	Chromosome	Length (Mb)	GC%
OX383284.1	1	30.92	37.5
OX383285.1	2	29.37	37.5
OX383286.1	3	29.21	37.0
OX383287.1	4	29.12	37.0
OX383288.1	5	28.83	37.0
OX383289.1	6	28.71	37.0
OX383290.1	7	28.67	37.5
OX383291.1	8	28.62	37.5
OX383292.1	9	27.78	37.5
OX383293.1	10	27.55	37.0
OX383294.1	11	27.13	37.0
OX383295.1	12	26.86	37.0
OX383296.1	13	26.64	37.5
OX383297.1	14	26.44	37.0
OX383298.1	15	25.72	37.5
OX383299.1	16	25.29	37.5
OX383300.1	17	25.17	37.5
OX383301.1	18	25.14	37.5
OX383302.1	19	24.94	37.5
OX383303.1	20	24.09	37.5
OX383304.1	21	22.36	37.0
OX383305.1	22	21.97	37.5
OX383306.1	23	21.64	37.5
OX383307.1	24	21.07	37.5
OX383308.1	25	18.43	37.5
OX383309.1	26	17.69	37.0
OX383310.1	27	13.55	37.5
OX383311.1	28	13.44	37.5
OX383312.1	29	12.97	38.5
OX383314.1	30	11.59	37.5
OX383313.1	W	12.36	39.0
OX383283.1	Z	33.33	37.0
OX383315.1	MT	0.02	19.0

The estimated Quality Value (QV) of the final assembly is 67.5 with *k*-mer completeness of 100%, and the assembly has a BUSCO v5.3.2 completeness of 99.0% (single = 98.5%, duplicated = 0.5%), using the lepidoptera_odb10 reference set ( *n* = 5,286).

Metadata for specimens, spectral estimates, sequencing runs, contaminants and pre-curation assembly statistics can be found at https://links.tol.sanger.ac.uk/species/2964684.

## Genome annotation report

The *Dryobota labecula* genome assembly (GCA_947523025.1) was annotated using the Ensembl rapid annotation pipeline ( Table 1; https://rapid.ensembl.org/Dryobota_labecula_GCA_947523025.1/Info/Index). The resulting annotation includes 19,099 transcribed mRNAs from 18,924 protein-coding genes.

## Methods

### Sample acquisition and nucleic acid extraction

The specimen selected for genome sequencing was a female *Dryobota labecula* (specimen ID Ox001958, individual ilDryLabe1), collected from Wytham Woods, Oxfordshire (biological vice-county Berkshire), UK (latitude 51.77, longitude –1.34) on 2021-10-09 using a light trap. The specimen was collected and identified by Peter Holland (University of Oxford) and snap-frozen on dry ice.

The ilDryLabe1 sample was prepared for DNA extraction at the Tree of Life laboratory, Wellcome Sanger Institute (WSI). The ilDryLabe1 specimen was weighed and dissected on dry ice with tissue set aside for Hi-C sequencing. Head and thorax tissue was disrupted using a Nippi Powermasher fitted with a BioMasher pestle. DNA was extracted at the WSI Scientific Operations core using the Qiagen MagAttract HMW DNA kit, according to the manufacturer’s instructions.

### Sequencing

Pacific Biosciences HiFi circular consensus DNA sequencing libraries were constructed according to the manufacturers’ instructions. DNA sequencing was performed by the Scientific Operations core at the WSI on the Pacific Biosciences SEQUEL II (HiFi) instrument. Hi-C data were also generated from ilDryLabe1 using the Arima2 kit and sequenced on the Illumina NovaSeq 6000 instrument.

### Genome assembly, curation and evaluation

Assembly was carried out with Hifiasm ( Cheng *et al.*, 2021) and haplotypic duplication was identified and removed with purge_dups ( Guan *et al.*, 2020). The assembly was then scaffolded with Hi-C data ( Rao *et al.*, 2014) using YaHS ( Zhou *et al.,* 2023). The assembly was checked for contamination and corrected as described previously ( Howe *et al.*, 2021). Manual curation was performed using HiGlass ( Kerpedjiev *et al.*, 2018) and Pretext ( Harry, 2022). The mitochondrial genome was assembled using MitoHiFi ( Uliano-Silva *et al.*, 2022), which runs MitoFinder ( Allio *et al.*, 2020) or MITOS ( Bernt *et al.*, 2013) and uses these annotations to select the final mitochondrial contig and to ensure the general quality of the sequence.

A Hi-C map for the final assembly was produced using bwa-mem2 ( Vasimuddin *et al.*, 2019) in the Cooler file format ( Abdennur & Mirny, 2020). To assess the assembly metrics, the *k*-mer completeness and QV consensus quality values were calculated in Merqury ( Rhie *et al.*, 2020). This work was done using Nextflow ( Di Tommaso *et al.*, 2017) DSL2 pipelines “sanger-tol/readmapping” ( Surana *et al.,* 2023a) and “sanger-tol/genomenote” ( Surana *et al.*, 2023b). The genome was analysed within the BlobToolKit environment ( Challis *et al.*, 2020) and BUSCO scores ( Manni *et al.*, 2021; Simão *et al.*, 2015) were calculated.

Table 3 contains a list of relevant software tool versions and sources.

**Table 3.** Software tools: versions and sources.

Software tool	Version	Source
BlobToolKit	4.1.5	https://github.com/blobtoolkit/blobtoolkit
BUSCO	5.3.2	https://gitlab.com/ezlab/busco
Hifiasm	0.16.1-r375	https://github.com/chhylp123/hifiasm
HiGlass	1.11.6	https://github.com/higlass/higlass
Merqury	MerquryFK	https://github.com/thegenemyers/MERQURY.FK
MitoHiFi	2	https://github.com/marcelauliano/MitoHiFi
PretextView	0.2	https://github.com/wtsi-hpag/PretextView
purge_dups	1.2.3	https://github.com/dfguan/purge_dups
sanger-tol/genomenote	v1.0	https://github.com/sanger-tol/genomenote
sanger-tol/readmapping	1.1.0	https://github.com/sanger-tol/readmapping/tree/1.1.0
YaHS	1.1a.2	https://github.com/c-zhou/yahs

### Genome annotation

The BRAKER2 pipeline ( Brůna *et al.*, 2021) was used in the default protein mode to generate annotation for the *Dryobota labecula* assembly (GCA_947523025.1) in Ensembl Rapid Release.

### Wellcome Sanger Institute – Legal and Governance

The materials that have contributed to this genome note have been supplied by a Darwin Tree of Life Partner. The submission of materials by a Darwin Tree of Life Partner is subject to the **‘Darwin Tree of Life Project Sampling Code of Practice’**, which can be found in full on the Darwin Tree of Life website here. By agreeing with and signing up to the Sampling Code of Practice, the Darwin Tree of Life Partner agrees they will meet the legal and ethical requirements and standards set out within this document in respect of all samples acquired for, and supplied to, the Darwin Tree of Life Project.

Further, the Wellcome Sanger Institute employs a process whereby due diligence is carried out proportionate to the nature of the materials themselves, and the circumstances under which they have been/are to be collected and provided for use. The purpose of this is to address and mitigate any potential legal and/or ethical implications of receipt and use of the materials as part of the research project, and to ensure that in doing so we align with best practice wherever possible. The overarching areas of consideration are:

- Ethical review of provenance and sourcing of the material
- Legality of collection, transfer and use (national and international)

Each transfer of samples is further undertaken according to a Research Collaboration Agreement or Material Transfer Agreement entered into by the Darwin Tree of Life Partner, Genome Research Limited (operating as the Wellcome Sanger Institute), and in some circumstances other Darwin Tree of Life collaborators.

## Data Availability

European Nucleotide Archive: *Dryobota labecula* (oak rustic). Accession number PRJEB57678; https://identifiers.org/ena.embl/PRJEB57678. ( Wellcome Sanger Institute, 2022) The genome sequence is released openly for reuse. The *Dryobota labecula* genome sequencing initiative is part of the Darwin Tree of Life (DToL) project. All raw sequence data and the assembly have been deposited in INSDC databases. Raw data and assembly accession identifiers are reported in Table 1.

## Author information

Members of the University of Oxford and Wytham Woods Genome Acquisition Lab are listed here: https://doi.org/10.5281/zenodo.4789928.

Members of the Darwin Tree of Life Barcoding collective are listed here: https://doi.org/10.5281/zenodo.4893703.

Members of the Wellcome Sanger Institute Tree of Life programme are listed here: https://doi.org/10.5281/zenodo.4783585.

Members of Wellcome Sanger Institute Scientific Operations: DNA Pipelines collective are listed here: https://doi.org/10.5281/zenodo.4790455.

Members of the Tree of Life Core Informatics collective are listed here: https://doi.org/10.5281/zenodo.5013541.

Members of the Darwin Tree of Life Consortium are listed here: https://doi.org/10.5281/zenodo.4783558.
