# Supplementary material for: The genome sequence of the Oak Rustic, Dryobota labecula (Esper, 1788)
Source: Wellcome Open Res. 2023 Jul 21;8:317. [Version 1] doi: 10.12688/wellcomeopenres.19541.1 (PMC11576569; doi:10.12688/wellcomeopenres.19541.1)
Supplement: Peer review history

# Reviewer report: Reviewer response for version 1

**DOI:** 10.21956/wellcomeopenres.21645.r104805

**Published:** 2024-11-19

**Authors:** Annabel Whibley

**Affiliations:** 1 The University of Auckland, Auckland, Auckland, New Zealand; 2 Bragato Research Institute, Lincoln, New Zealand

In this Data Note, Holland and colleagues present a reference genome assembly and gene annotation of the Oak Rustic ( *Dryobota labecula*). The reference is high-quality, and has been constructed using appropriate tools and with comprehensive reporting of the sample collection, data generation and analysis and all associated metadata. A clear description of the species, its natural history and potential avenues of future research are provided. Links to data accessions and interactive plots are functional.

Are sufficient details of methods and materials provided to allow replication by others?

Yes

Is the rationale for creating the dataset(s) clearly described?

Yes

Are the datasets clearly presented in a useable and accessible format?

Yes

Are the protocols appropriate and is the work technically sound?

Yes

Reviewer Expertise:

Bioinformatics, Genomics

I confirm that I have read this submission and believe that I have an appropriate level of expertise to confirm that it is of an acceptable scientific standard.

# Reviewer report: Reviewer response for version 1

**DOI:** 10.21956/wellcomeopenres.21645.r104799

**Published:** 2024-10-16

**Authors:** José M Martín-Durán

**Affiliations:** 1 Queen Mary University of London, London, UK

This Genome Note reports the sequencing and assembly of the genome of the moth *Dryobota labecula*. This is a common moth in the Mediterranean basin whose geographical range appears to expand north, perhaps due to climate warming. This might have significant consequences for the existing moth biodiversity in the UK and oaks, as the larvae appear to feed on these trees. A high-quality genomic resource is, therefore, an important resource to investigate the adaptive mechanisms in these moths.

**Strengths:**

 - High-quality genome assembly and annotation.

 - Sequencing and analytical pipelines follow standard procedures, which will facilitate comparative analyses with other genomes and species.

**Weaknesses:**

 - None. This is a high-quality genomic resource that will enable multiple research avenues.

Are sufficient details of methods and materials provided to allow replication by others?

Yes

Is the rationale for creating the dataset(s) clearly described?

Yes

Are the datasets clearly presented in a useable and accessible format?

Yes

Are the protocols appropriate and is the work technically sound?

Yes

Reviewer Expertise:

Comparative genomics, invertebrate development and evolution

I confirm that I have read this submission and believe that I have an appropriate level of expertise to confirm that it is of an acceptable scientific standard.
